# Establishment of a pancreatic stem cell line from fibroblast-derived induced pluripotent stem cells

**DOI:** 10.1186/1475-925X-13-64

**Published:** 2014-05-27

**Authors:** Takashi Kuise, Hirofumi Noguchi, Hiroshi Tazawa, Takashi Kawai, Masaya Iwamuro, Issei Saitoh, Hitomi Usui Kataoka, Masami Watanabe, Yasufumi Noguchi, Toshiyoshi Fujiwara

**Affiliations:** 1 Department of Gastroenterological Surgery, Okayama University Graduate School of Medicine, Dentistry and Pharmaceutical Sciences, Okayama 700-8558, Japan; 2 Department of Surgery, Chiba-East National Hospital, National Hospital Organization, Chiba 260-8712, Japan; 3 Department of Gastroenterology and Hepatology, Okayama University Graduate School of Medicine, Dentistry, and Pharmaceutical Sciences, Okayama 700-8558, Japan; 4 Department of Pediatric Dentistry, Niigata University Graduate School of Medical and Dental Sciences, Niigata 951-8514, Japan; 5 Department of Primary Care and Medical Education, Okayama University Graduate School of Medicine, Dentistry and Pharmaceutical Sciences, Okayama 700-8558, Japan; 6 Department of Urology, Okayama University Graduate School of Medicine, Dentistry and Pharmaceutical Sciences, Okayama 700-8558, Japan; 7 Department of Socio-environmental Design, Hiroshima International University, Hiroshima 737-0112, Japan

**Keywords:** Mouse pancreatic stem cells, Diabetes, iPS cells

## Abstract

**Background:**

For cell therapies to treat diabetes, it is important to produce a sufficient number of pancreatic endocrine cells that function similarly to primary islets. Induced pluripotent stem (iPS) cells represent a potentially unlimited source of functional pancreatic endocrine cells. However, the use of iPS cells for laboratory studies and cell-based therapies is hampered by their high tumorigenic potential and limited ability to generate pure populations of differentiated cell types *in vitro*. The purpose of this study was to establish a pancreatic stem cell line from iPS cells derived from mouse fibroblasts.

**Methods:**

Mouse iPS cells were induced to differentiate into insulin-producing cells by a multi-step differentiation protocol, which was conducted as described previously with minor modifications. Selection of the pancreatic stem cell was based on morphology and Pdx1 expression. The pancreatic potential of the pancreatic stem cells was evaluated using a reverse transcription PCR, real-time PCR, immunofluorescence, and a glucose challenge test. To assess potential tumorigenicity of the pancreatic stem cells, the cells were injected into the quadriceps femoris muscle of the left hindlimb of nude mice.

**Results:**

The iPS-derived pancreatic stem cells expressed the transcription factor –Pdx1– a marker of pancreatic development, and continued to divide actively beyond passage 80. Endocrine cells derived from these pancreatic stem cells expressed insulin and pancreatic genes, and they released insulin in response to glucose stimulation. Mice injected with the pancreatic stem cells did not develop tumors, in contrast to mice injected with an equal number of iPS cells.

**Conclusion:**

This strategy provides a new approach for generation of insulin-producing cells that is more efficient and safer than using iPS cells. We believe that this approach will help to develop a patient-specific cell transplantation therapy for diabetes in the near future.

## Background

Production of a sufficient number of insulin-producing cells from stem cells that function similarly to primary islets is important for clinical application of stem cell therapy to diabetes. Many studies have reported the differentiation of insulin-producing cells from mouse embryonic stem (ES) cells and, more recently, from human ES cells [1-7]. Unfortunately, these methods involving ES cells have various limitations such as ethical issues during the generation of the cells and immunological rejection after an allogeneic transplant. Induced pluripotent stem (iPS) cell technology has the potential to generate patient-specific cell types including functional pancreatic endocrine cells [8-11]. However, the use of ES and iPS cells for laboratory studies and cell-based therapies is hampered by their high tumorigenic potential and limited ability to generate pure populations of differentiated cell types *in vitro*.

D’Amour *et al.* developed a 5-step protocol for differentiation of human ES cells into pancreatic hormone-expressing cells in 2006 [12]; this method represented a great step forward in regenerative medicine; however, the use of ES cells in clinical practice is problematic, as explained above. We and other groups have established mouse pancreatic stem cell lines using specific culture conditions [13-15]. We have also demonstrated that young mice have a high number of pancreatic stem cells that can be isolated, but older mice have a low number of pancreatic stem cells, and therefore are unable to provide viable clones [16]. Similarly, human pancreatic stem cells cannot be isolated from 20- to 60-year-old donors [17].

In this study, we established a pancreatic stem cell line from mouse iPS cells, which have the potential for self-renewal and multipotency to generate both endocrine and exocrine pancreatic cells.

## Methods

### Culture conditions

Mouse iPS cells (iPS-MEF-Ng-20D-17) were provided by the RIKEN BRC through the Project for Realization of Regenerative Medicine and the National Bio-Resource Project of MEXT, Japan [18]. Undifferentiated iPS cells were maintained on mouse embryo fibroblast feeder layers (STO cell line) in Dulbecco’s modified Eagle medium (DMEM; Sigma-Aldrich, St Louis, MO, USA) supplemented with 15% (vol/vol) fetal bovine serum (FBS; Millipore, Billerica, MA, USA), 1% nonessential amino acids (Millipore), 1% nucleosides (Millipore), 1% penicillin/streptomycin (Sigma-Aldrich), 110 μM 2-mercaptoethanol (Life Technologies, Tokyo, Japan), and 500 U/mL leukemia inhibitory factor (LIF; Millipore) at 37°C. Cultures were manually passaged at a 1:4–1:8 split ratio every 3–5 days.

**Figure 1:**
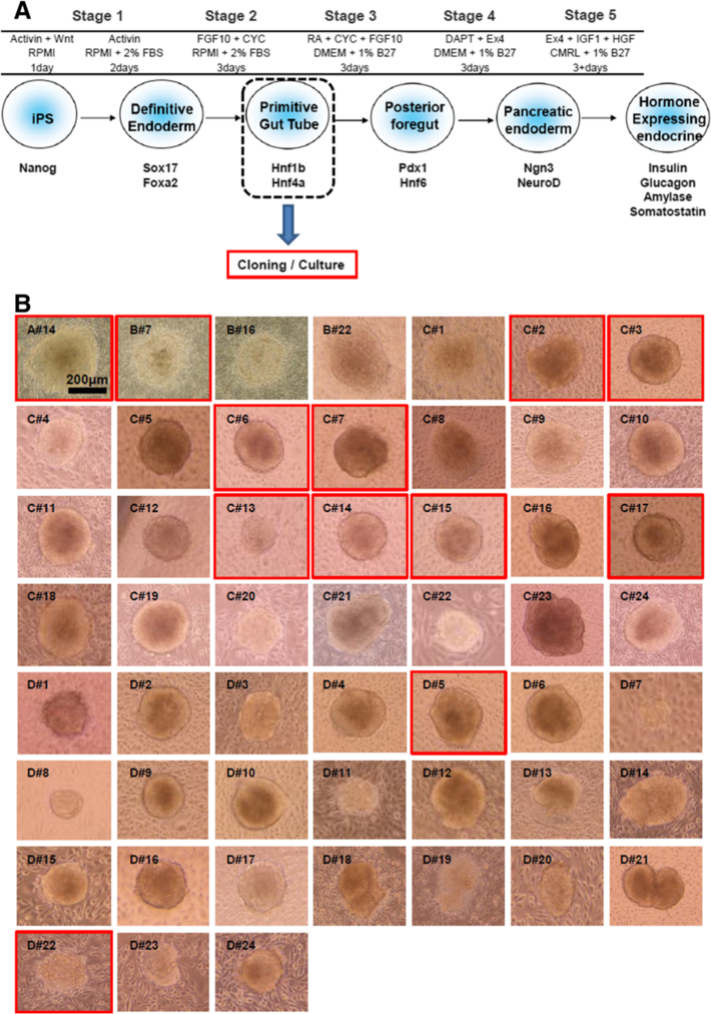
**A five-step protocol for differentiation of iPS cells to pancreatic stem cells and hormone-expressing cells. A**. Schematic representation of the differentiation procedure and protein expression of key markers of pancreatic differentiation. Based on D’Amour’s 5-step protocol [12], this differentiation protocol is subdivided into 5 stages, and the growth factors, medium, and range of duration of each stage are shown. Several markers characteristic of each cell population are listed. Pancreatic stem cells were established after induction of stages 1 and 2. Abbreviations: CYC, KAAD-cyclopamine; RA, all-*trans*-retinoic acid; DAPT, γ-secretase inhibitor; Ex4, exendin-4; iPS, induced pluripotent stem cell; ME, mesendoderm; DE, definitive endoderm; PG, primitive gut tube; PF, posterior foregut endoderm; PE, pancreatic endoderm and endocrine precursor; EN, hormone-expressing endocrine cells. **B**. Morphology of 52 clones after induction of stages 1 and 2 (passage 2). Scale bars = 200 μm.

Directed differentiation into insulin-producing cells was conducted as described previously [12], with minor modifications (Figure 1). At stage 1, cells were incubated with 25 ng/mL Wnt3a and 100 ng/mL activin A (R&D Systems, Minneapolis, MN, USA) in the RPMI medium (Life Technologies) at 37°C for 1 day, followed by treatment with 100 ng/mL activin A in RPMI (containing 0.2% FBS) at 37°C for 2 days. At stage 2, the cells were incubated with 50 ng/mL FGF10 (R&D Systems) and 0.25 μM KAAD-cyclopamine (Toronto Research Chemicals, Toronto, Ontario, Canada) in RPMI (containing 2% FBS) at 37°C for 3 days. At stage 3, the cells were incubated with 50 ng/mL fibroblast growth factor 10 (FGF10), 0.25 μM KAAD-cyclopamine, and 2 μM all-*trans* retinoic acid (Sigma-Aldrich) in DMEM with a 1% (vol/vol) B27 supplement (Life Technologies) at 37°C for 3 days. At stage 4, the cells were treated with 1 μM N-[N-(3,5-Difluorophenacetyl)-L-alanyl]-S-phenylglycine t-butyl ester (DAPT; Sigma-Aldrich) and 50 ng/mL exendin-4 (Sigma-Aldrich) in DMEM with a 1% (vol/vol) B27 supplement at 37°C for 3 days. At stage 5, the cells were incubated with 50 ng/mL exendin-4, 50 ng/mL IGF-1 (Sigma), and 50 ng/mL hepatocyte growth factor (HGF; R&D Systems) in the CMRL medium (Life technologies) with a 1% (vol/vol) B27 supplement at 37°C for 3–6 days.

### Tumorigenesis assay

To examine the potential tumorigenicity of candidate clones at passage 50, 1 × 10^7^ cells were injected into the quadriceps femoris muscle of the left hindlimb of nude mice (n = 3). As a positive control, we transplanted 1 × 10^7^ iPS cells into the right hindlimb. All mouse studies were approved by the Institutional Animal Care and Use Committee of Okayama University (Reference number: OKU-2011351).

### Semi-quantitative RT-PCR

Total RNA was extracted from cells using the RNeasy Mini Kit (Qiagen, Tokyo, Japan). After the RNA was quantified using spectrophotometry, 2.5 μg of the RNA was heated at 85°C for 3 min and then reverse-transcribed into cDNA in a 25-μL reaction containing 200 units of Superscript III RT (Life Technologies), 50 ng of random hexamer primers (Life Technologies), 160 μmol/L dNTP, and 10 nmol/L dithiothreitol. The reaction consisted of 10 min at 25°C, 60 min at 42°C, and 10 min at 95°C. PCRs were performed in a Perkin-Elmer 9700 Thermocycler with 3 μL of cDNA (20 ng RNA equivalent), 160 μmol/L cold dNTPs, 10 pmol of the appropriate oligonucleotide primers, 1.5 mmol/L MgCl_2_, and 5 units of AmpliTaq Gold DNA polymerase (Perkin-Elmer, Waltham, MA, USA). The oligonucleotide primers and cycle numbers used for semi-quantitative PCR are shown in Table 1. The thermal cycle profile used a 10-min denaturing step at 94 C followed by the amplification cycles (1 min denaturation at 94 C, 1 min annealing at 57 C, and 1 min extension at 72°C), with a final extension step of 10 min at 72°C. The steps taken to validate these measurements were previously reported [19].

**Table 1:** List of gene-specific primers

**Gene**	**Forward/Reverse primer (5′ → 3′)**
Nanog	cacaggctctttcttcagattg/tcttgcttgctcttcacattgg
Pdx1	cggacatctccccatacg/aaagggagctggacgcgg
Insulin-2	tccgctacaatcaaaaaccat/gctgggtagtggtgggtcta
Glucagon	agaagggcagagcttgggcc/tgctgcctggccctccaagt
Amylase	tggccttctggatcttgc/aaaggtctgcttccttggg
Somatostatin	atgctgtcctgccgtctc/ttctctgtctggttgggctc
Gapdh	accacagtccatgccatcac/tccaccaccctgttgctgta

### TaqMan real-time PCR

Quantification of Ngn3, NeuroD, and insulin-2 mRNA levels was conducted using the TaqMan real-time PCR system according to the manufacturer’s instructions (Life Technologies). PCR consisted of 40 cycles including 2 min at 50°C and 10 min at 95°C as initial steps. In each cycle, denaturation was performed for 15 s at 95°C and annealing/extension was 1 min at 60°C. PCR was conducted in 20-μL reaction containing cDNA synthesized from 1,500 ng of total RNA. Standard curves were constructed using cDNA generated from total RNA isolated from primary mouse islets. For each sample, the expression of Ngn3, NeuroD, and insulin-2 was normalized against the β-actin expression level. Mouse Ngn3, NeuroD, and insulin-2 and β-actin primers were obtained commercially (Assays-on-Demand Gene Expression Products; Life Technologies).

### The glucose challenge

To remove insulin added to the culture medium, cultured cells were washed 10 times with phosphate-buffered saline (PBS). The RPMI medium with a low glucose concentration (2.8 mM) was added, and the cells were cultured at 37°C for 30 min as preincubation. After the preincubation, RPMI with a low glucose concentration (2.8 mM) was added, and the cells were cultured at 37°C for 60 min. This low-glucose medium was then collected. The RPMI medium with a high glucose concentration (20 mM) was then added, and the cells were cultured at 37°C for 60 min. This high-glucose medium was also collected. The collected media were assayed for insulin concentration using an enzyme-linked immunosorbent assay (ELISA; SRL, Tokyo, Japan).

### An immunofluorescence assay

Cells were fixed in 4% paraformaldehyde for 20 min. Subsequently, the cells were blocked with 10% serum and 0.2% Triton X-100 in PBS, and then incubated with a primary antibody against mouse insulin (guinea pig polyclonal antibody to mouse insulin, 1:100; Abcam, Tokyo, Japan) overnight at 4°C. The cells were next incubated with a secondary antibody (goat polyclonal antibody against guinea pig IgG, H&L labeled with fluorescein isothiocyanate [FITC], 1:100; Abcam). A medium for fluorescence microscopy that contained 4′,6-diamidino-2-phenylindole (DAPI; Vector Laboratories, Burlingame, CA, USA) was used for mounting onto slides.

## Results

### Directed pancreatic differentiation of fibroblast-derived iPS cells

**Figure 2:**
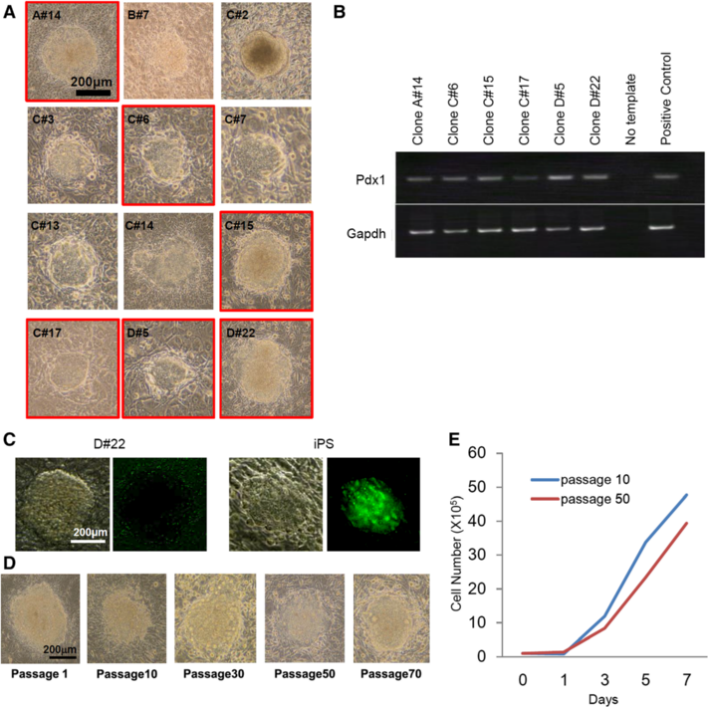
**Morphology and expression of Pdx1 and Nanog, and growth activity. A**. Morphology of 12 clones after 30-day culture (passage 7). Scale bars = 200 μm. **B**. Pdx1 gene expression of 6 candidate clones. These 6 clones were selected based on their ability to grow in culture for over 30 days. Pancreatic stem cells from pancreatic tissue (data not shown) were used as a positive control. **C**. Nanog (GFP) expression of D#22 and iPS cells. The iPS cells but not D#22 cells expressed GFP. **D**. Morphology of D#22 cells at several passages. Scale bars = 200 μm. **E**. Growth activity of D#22 cells at passages 10 and 50.

To generate pancreatic stem cells from iPS cells, we applied the D’Amour’s protocol [12]. At stage 1, iPS cells differentiated into definitive endoderm using high concentrations of activin A with Wnt3a (day 1) and FBS supplementation (days 2–3). At stage 2, the definitive endoderm cells differentiated into gut tube endoderm through removal of activin A and addition of FGF10 and KAAD-cyclopamine. After the cells were passaged once and cultured for 4 days, the clones were manually picked under a dissecting microscope (Figure 1A). These clones were then cultured on STO feeder cells in the ES culture medium. The 52 clones were cultured and passaged for 30 days (Figure 1B). After the 30-day culture of these clones, 12 clones were still viable (Figure 2A),

### Establishment of pancreatic stem cells

Among the 12 clones, 6 were eliminated because they did not proliferate as actively as did the iPS cells. Expression of pancreatic and duodenal homeobox factor 1 (Pdx1) mRNA, a transcription factor required for pancreatic development and β-cell maturation, in the remaining 6 clones is shown in Figure 2B. The proliferative activity of 4 of the 6 clones significantly decreased during a 60-day culture after the isolation. Therefore, we selected the 2 clones (C#15 and D#22) that maintained a constant cell proliferation rate as candidate clones for differentiation into pancreatic stem cells. Clone C#15 was ascertained to have a tumorigenic potential. Finally, clone D#22 was selected (Figure 2C). D#22 cells did not express green fluorescent protein (GFP), whereas the iPS cells in this study expressed GFP according to Nanog expression [18]; this result suggests that D#22 cells do not express Nanog and are therefore different from iPS cells (Figure 2C).

### Morphology, gene expression, and growth activity of the pancreatic stem cell line D#22

**Figure 3:**
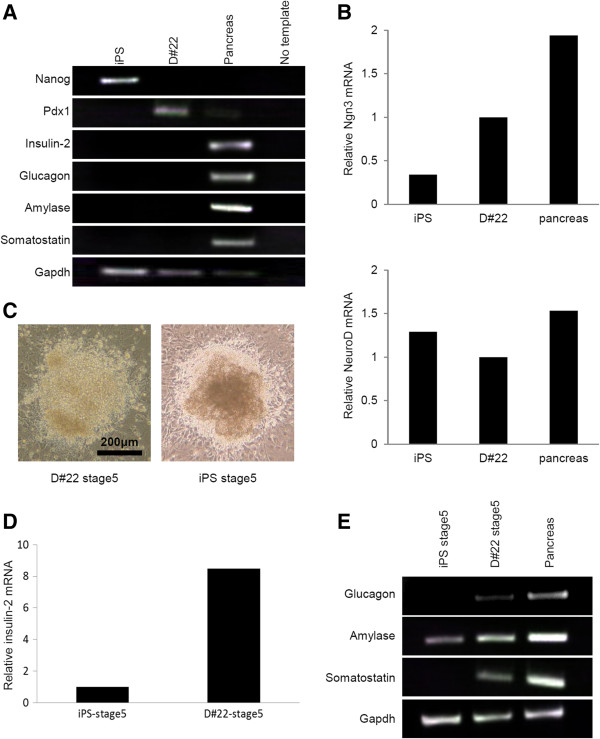
**Characterization of the pancreatic stem cell line D#22. A**. RT-PCR analysis of expression of an ES cell marker (Nanog), pancreatic progenitor markers (Pdx1), and mature pancreatic markers (insulin-2, glucagon, amylase, and somatostatin) in D#22 cells. Pancreatic cells and iPS cells were used as controls. The primers used for RT-PCR are shown in Table 1. **B**. Quantitative RT-PCR analysis of the Ngn3 and NeuroD genes in iPS, D#22, and pancreatic cells. The data are expressed as the Ngn3 and NeuroD to β-actin ratio, with the expression in D#22 cells arbitrarily set at 1.0. **C**. Morphology of D#22 stage 5 cells and iPS stage 5 cells. Scale bars = 200 μm. **D**. Quantitative RT-PCR analysis of the insulin-2 gene in D#22 stage 5 cells and iPS stage 5 cells. Differentiated cells derived from D#22 cells by stages 3–5 or iPS cells by stages 1–5 were analyzed using quantitative RT-PCR. The data are expressed as the insulin-2 to β-actin ratio, with the expression in iPS stage 5 cells arbitrarily set at 1.0. **E**. RT-PCR testing for pancreatic markers of maturity (glucagon, amylase, and somatostatin) in D#22 stage 5 and iPS stage 5 cells. Pancreatic cells were used as a control. The primers used for RT-PCR are shown in Table 1.

Clone D#22 formed a flat “cobblestone” monolayer, which is characteristic of cultured duct cells [20]. D#22 cells continued to divide actively beyond passage 80 (over 6 months) without changes in morphology (Figure 2D) or in growth activity (Figure 2E). We performed normal and quantitative RT-PCR testing of clone D#22 for ES cell markers, endodermal/pancreatic progenitor cell markers, and pancreatic cell markers. Mouse pancreas cDNA and iPS cells were used as controls. Representative genes of the posterior foregut (Pdx1) and pancreatic endoderm (Ngn3, NeuroD) were expressed by clone D#22, whereas genes typically expressed in undifferentiated iPS cells (Nanog) and in pancreatic tissue (insulin, glucagon, amylase, and somatostatin) were not expressed in D#22 (Figure 3A, B).

### The differentiation capacity of the pancreatic stem cell line D#22

For differentiation of D#22 cells into insulin-producing cells, the following protocols were used (stages 3–5 in Figure 1A). At the final stage of differentiation, D#22 cells underwent a morphological transition (Figure 3C). The differentiated cells derived from D#22 cells, named D#22 stage 5 cells, were tested for pancreas-specific gene expression and insulin production. Mouse iPS cells were also induced to differentiate using the 5-step protocol (Figure 1); the pancreatic potential of the final product (iPS stage 5 cells) was compared with that of D#22 stage 5 cells. Quantitative PCR demonstrated that levels of insulin 2 mRNA were 8.46-fold higher in D#22 stage 5 cells than in iPS stage 5 cells (Figure 3D). D#22 stage 5 cells were positive for gene expression of hormones produced in the endocrine pancreas (e.g*.*, insulin, glucagon, and somatostatin) in addition to a representative enzyme produced in the exocrine pancreas (amylase; Figure 3E).

### Insulin expression and functional analysis of D#22 stage 5 cells

**Figure 4:**
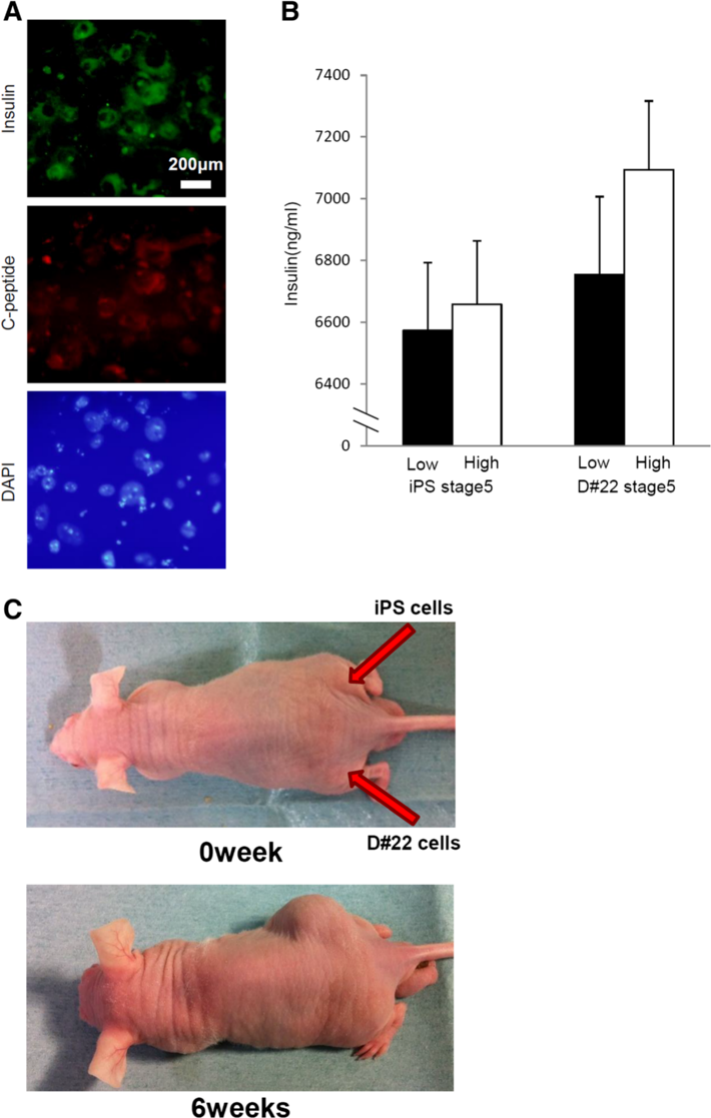
**Insulin expression and teratoma formation/tumorigenicity assay of the pancreatic stem cell line D#22. A**. Immunofluorescence analysis of differentiated cells derived from D#22 cells. Insulin and C-peptide staining of D#22 stage 5 cells was performed. Scale bars = 200 μm. **B**. Insulin release assay. D#22 stage 5 cells and iPS stage 5 cells were stimulated with D-glucose at 2.8 mM and 20 mM, and the amount of insulin released into the culture supernatant was measured using ELISA. Error bars represent standard error. **C**. In total, 1 × 10^7^ of D#22 cells were injected into the thigh of nude mice. As a positive control, we transplanted 1 × 10^7^ iPS cells into the contralateral thigh.

To determine whether D#22 cells could differentiate into insulin-producing cells, immunofluorescence analysis was performed. Some D#22 stage 5 cells were positive for insulin, and the insulin-positive cells were C-peptide positive, thus excluding the possibility of insulin uptake from the medium (Figure 4A).

To evaluate the insulin production of D#22 stage 5 cells, a glucose challenge test was performed *in vitro*. The amount of insulin secreted by D#22 stage 5 cells tended to increase when the glucose concentration of the medium was high, whereas iPS stage 5 cells showed little or no response to the glucose challenge (Figure 4B). These data suggested that D#22 stage 5 cells had the ability to secrete insulin in response to the pathological glucose concentrations similar to those in diabetic patients.

### Tumorigenicity of the pancreatic stem cell line D#22

To rule out the possibility of teratoma formation as a result of contamination with iPS cells or spontaneous transformation of D#22 cells, the tumorigenic potential was tested in vivo. D#22 cells (1 × 10^7^) at passage 50 were transplanted into nude mice (n = 3), and no tumors developed after 2 months. In contrast, injection of 1 × 10^7^ iPS cells resulted in tumor development after 2 weeks. Tumors derived from the iPS cells became larger over time and had a size of 40 mm at 2 months after transplantation (Figure 4C).

## Discussion

In this study, we established a pancreatic stem cell line, D#22, from mouse fibroblast-derived iPS cells. Generally, stem cells are defined as cells capable of self-renewal that can develop into various lineages in the body. D#22 cells were maintained by repeated passaging for more than 6 months without growth inhibition, which indicates that D#22 cells have the potential for self-renewal. The differentiated D#22 stage 5 cells express insulin, glucagon, amylase, and somatostatin mRNA; these data indicate that D#22 cells can generate both endocrine and exocrine pancreatic lineages. Consequently, we consider the clone D#22 a pancreatic stem cell line. As shown in Figure 3A, D#22 cells express Pdx1, Ngn3, and NeuroD. Pdx1-expressing epithelial progenitors are involved in the development of organs during embryogenesis, especially the cells giving rise to the endocrine, exocrine, and ductal cells of the pancreas [21]. Ngn3 is expressed in all endocrine progenitors [21], initiating a cascade of expression of transcription factors that control endocrine cell differentiation. NeuroD, a basic helix-loop-helix (bHLH) transcription factor, is also a key regulator of pancreatic islet morphogenesis and insulin gene transcription [22,23]. However, D#22 cells did not express key transcription factors of ES cells (Nanog) or mature pancreatic cells (insulin, glucagon, amylase, and somatostatin). These data indicate that D#22 cells have the characteristics of pancreatic endoderm-committed intermediates or pancreatic progenitors.

D#22 stage 5 cells secreted insulin in response to glucose stimulation, and immunofluorescent analysis of the differentiated cells showed production of insulin and C-peptide. Moreover, in the real-time PCR analysis, mRNA expression of insulin 2 was 8.46-fold higher in D#22 stage 5 cells than in iPS stage 5 cells generated using the same stepwise differentiation protocol. In addition, clone D#22 is not tumorigenic, whereas transplanted iPS cells rapidly form tumors. In general, tumorigenesis is a major concern in the clinical application of stem cell therapy. Therefore, we can conclude that D#22 cells are potentially a more useful cell source for β-cell replacement therapy in diabetes than iPS cells because the former are safer and capable of generating greater numbers of functional insulin-producing cells. Nevertheless, the level of insulin expression was still low; suggesting that the induced cells were still immature. Therefore, it is necessary to develop a more efficient differentiation protocol for production of insulin-producing cells.

The advantages of D#22 cells compared with iPS cells are 1) more efficient differentiation, and 2) no teratoma formation. Recently, a self-renewing endodermal progenitor (EP) cell line generated from human ES and iPS cells was reported [24]. The self-renewing EP cells display morphological properties and a gene expression pattern characteristic of a definitive endoderm. These EP cells differentiate into pancreatic cells, hepatocytes, and intestinal epithelial cells. Moreover, EP cells are not tumorigenic in vivo. D#22 cells may be similar to EP cells, but are likely to be differentiated further into the pancreatic linage because they express the Pdx1, Ngn3, and NeuroD transcription factors.

## Conclusions

We established a mouse pancreatic stem cell line from iPS cells derived from mouse embryonic fibroblasts. This clonal cell line has the ability of self-renewal and efficiently differentiates into insulin-producing cells without any signs of tumorigenesis. Therefore, this strategy provides a new approach for generation of insulin-producing cells more efficiently and safely compared to iPS cells. We believe that this approach will help implement a patient-specific cell transplantation therapy for diabetic patients in the near future.

## Abbreviations

DAPI: 4′,6-diamidino-2-phenylindole; DAPT: N-[N-(3,5-Difluorophenacetyl)-L-alanyl]-S-phenylglycine t-butyl ester; DMEM: Dulbecco’s modified Eagle medium; ES: Embryonic stem, FBS, fetal bovine serum; FGF10: Fibroblast growth factor 10; FITC: Fluorescein isothiocyanate; Foxa2: Forkhead box protein a2; GFP: Green fluorescent protein; HGF: Hepatocyte growth factor; IGF-1: Insulin-like growth factor 1; iPS: Induced pluripotent stem; LIF: Leukemia inhibitory factor; MEXT: Ministry of Education, Culture, Sports, Science, and Technology of Japan; Pdx1: Pancreatic and duodenal homeobox factor 1.

## Competing interests

The authors declare that they have no competing interests.

## Authors’ contributions

TK carried out most of the experimental work with the help of. HT, TK, MI. HN designed the experiments and analyzed the data. IS, HUK, MW, YN and TF provided materials and discussion. TK and HN wrote the manuscript. All authors discussed and commented on the manuscript. All authors read and approved the final manuscript.
